# Supplementary material for: Non-Targeted Metabolomics Reveals the Effects of Different Rolling Methods on Black Tea Quality
Source: Foods. 2024 Jan 19;13(2):325. doi: 10.3390/foods13020325 (PMC10815122; doi:10.3390/foods13020325)
Supplement: Supplementary file 1 [file foods-13-00325-s001.zip › foods-2801846-supplementary.pdf]

Supplementary Materials

Table S1. List of abbreviations.

Abbreviation	Full name	Abbreviation	Full name
GC-MS	Gas chromatography-Mass spectrometry	UHPLC-Q-TOF/MS	Ultra-High Performance Liquid Chromatography-Quadrupole Time-of-flight Mass Spectrometry
GBVs	green bitterness volatiles	PPO	polyphenol oxidase
POD	peroxidase	HS-SPME	Head space-Solid phase micro-extraction
RI	Retention Index	PCA	Principal component analysis
OPLS-DA	Orthogonal partial least square discriminate analysis	ANOVA	Analysis of Variance
C	Catechin	CG	Catechin gallate
EC	Epicatechin catechin	ECG	Epicatechin gallate
EGCG	Epigallocatechin gallate	GC	Gallo-catechin
GCG	Gallo-catechin gallate	TF	Theaflavin

Table S2. Detail information of Tea Samples.

Different rolling time processing (Process Sample)	Fresh leaves (FL)
	Rolled for 0min (RT0)
	Rolled for 20min (RT20)
	Rolled for 40min (RT40)
	Rolled for 60min (RT60)
	Rolled for 80min (RT80)
	Rolled for 100min (RT100)
	Rolled for 120min (RT120)

Table S3. Information of chemicals and reagents**.**

**Chemicals and reagents**	**Manufacturer**
Methanol of LC–MS grade	Thermo Fisher
LC-MS-grade formic acid, dichloromethane, n-alkanes C_3-C_{25}, etofylline, ethyl caprate	Sigma (St Louis, MO, USA)
Anhydrous sodium sulfate (Na_2SO_4) and sodium chloride (NaCl)	Shanghai Hushi Co., Ltd. (Shanghai, China)
Taste standards: amino acid standards (histidine, tryptophan, lysine, serine, glutamate, glutamine, aspartic acid, asparagine, arginine, tyrosine, proline, valine, isoleucine, leucine, threonine, phenylalanine, glycine, alanine, methionine, theanine, *γ*-aminobutyric acid), catechin standards (gallocatechin, epigallocatechin, catechin, epicatechin, epigallocatechin gallate, gallocatechin gallate, epicatechin gallate, epiafzelechin, procyanidin B1/B2), theaflavin standards (theaflavin, theaflavin-3-gallate, theaflavin-3′-gallate, theaflavine-3,3′-digallate), phenolic acid standards (quinic acid, p-coumaric acid, chlorogenic acid, gallic acid, caffeic acid), alkaloid standards (theobromine, theophylline, caffeine), and flavonoid standards (vitexin glucoside, vitexin, vitexin rhamnoside, quercetin, quercetin-7-*O*-glucoside, quercetin 3-*O*-rutinoside, quercetin, quercetin-3-*O*-glucosylrhamnoside, quercetin-7-*O*-*α*-l-rhamnoside, quercetin-3-galactoside, quercetin-3-*O*-*β*-*d*-glucopyranoside, myricetin, myricetin-3-*O*-galactoside, kaempferol, dihydro kaempferol-7-*O*-rhamnoside, kaempferol-3-*O*-glucoside, isovitexin, isovitexin-2″-*O*-arabinoside).	Chemfaces (Wuhan, China)<br>Yuanye Bio-Technology Co., Ltd (Shanghai, China)
GBVs: benzyl *β*-glucoside, 2-phenylethyl glucoside, (*Z*)-3-hexenyl glucoside, geranyl glucoside, benzyl β-primeveroside, nerol glucoside, 2-phenylethyl primeveroside, (*Z*)-3-hexenyl primeveroside, geranyl primeverosidea, nerol primeveroside	National Glycoengineering Research Center, NGRC (Jinan, China)
Aroma standards: 1-octen-3-ol, *p*-Cymene, cycloheptanol, 1-undecyne, n-decanoic acid, dimethyl phthalate, ethyl vanillin, *β*-ocimene, nonanal, benzyl acetate, (*Z*)-2-hexenyl hexanoate, myrtenol, dodecane, decanal, (*E*,*E*)-2,4-nonadienal, citronellol, (*Z*)-3-hexenyl-α-methylbutyrate, (*E*)-2-decenal, 2,6,11-trimethyl-dodecane, geranyl formate,, benzyl butyrate, geranyl acetate, tetradecane, *α*-farnesene, hexanoic acid phenylmethyl ester, methyl jasmonate, neophytadiene, 2-methyl-butanal, 3-methyl-3-buten-2-one, 1-penten-3-one, 2-methyl-3-buten-2-ol, undecane, 3-pentanol, (*E*)-2-pentenal, 1-penten-3-ol, *β*-myrcene, 3-methyl-1-	

butanol, (*E*)-2-hexenal, 1-pentanol, 1-hexanol, 2-cyclopenten-1-one, (*Z*)-3-hexen-1-ol, (*E*)-2-hexen-1-ol, tridecane, (*Z*)-2-hexen-1-ol, 1-heptanol, furfural, (*E*)-butanoic acid 3-hexenyl ester, linalool, (*Z*)-linalool oxide (furanoid), 1-octanol, dimethyl sulfoxide, hexadecane, (*E*)-2-octen-1-ol, 2,5-dimethylcyclohexanol, 1-ethyl-1H-pyrrole-2-carboxaldehyde, ethyl caprate, 1-nonanol, (*Z*)-hexanoic acid 3-hexenyl ester, (*Z*)-3-nonen-1-ol, *α*-terpineol, heptadecane, geranylacetone, maltol, 1-(1H-pyrrol-2-yl)-ethanone, 1-dodecanol, (3*Z*)-3-hexen-1-yl benzoate, 2,4-di-tert-butylphenol, coumarin, benzeneacetaldehyde, linalool oxide (pyranoid), naphthalene, geraniol, indole, α-ionone, β-phenylethyl butyrate, (*E*)-β-famesene, (*E*)-*β*-Ionone, dihydroactinidiolide, (*E*,*Z*)-2,6-nonadienal, (*E*,*E*)-2,4-heptadienal	Yuanye Bio-Technology Co., Ltd (Shanghai, China)<br><br>Aladdin Biochemical Technology Co., Ltd (Shanghai, China)<br><br>Sigma (St Louis, MO, USA)<br><br>MackLin Biochemical Technology Co., Ltd(Shanghai, China)
Color standards: Chlorophyll a and b, lutein, and β-carotene	Sigma (St Louis, MO, USA)

Table S4: Relative content of main volatile components during different rolling times of black tea (μg/g).

Compound	Compound name	R.T.	Actual RI	Reference RI	Relative content（μg/g）						
					RT0	RT20	RT40	RT60	RT80	RT100	RT120
Alcohols	3-Hexen-1-ol	4.14	864	856	5.18±1.37b	10.06±1.02a	9.79±1.79a	10.48±0.84a	10.16±1.6a	10.21±1.49a	10.72±1.37a
	cis-2-Hexen-1-ol	4.33	872	868	10.03±0.47d	20.66±1.27a	17.71±1.14b	17.07±0.76b	14.24±1.46c	13.02±0.51c	11.25±0.78d
	benzyl alcohol	6.19	959	962	24.44±1.14c	32.26±0.82b	29.85±1.68b	30.46±0.45b	31.2±2.09b	37.79±6.72a	32.91±1.65ab
	(cis)-Linalool oxide (furans)	9.32	1067	1074	25.98±2.35c	33.1±1.28a	30.81±1.55ab	30.79±1.68ab	29.24±1.45bc	26.98±2.98c	28.69±1.17bc
	(trans)-Linalool oxide (furans)	9.90	1085	1086	32.85±0.48c	44.73±2.44a	42.65±4.05ab	42.5±4.97ab	40.69±3.2ab	39.34±1.86b	39.17±0.91b
	linalool	10.39	1100	1099	35.45±2.64c	41.41±4.95ab	46.06±1.18a	41.57±1.06ab	44.09±1.58a	37.41±4.53bc	35.77±2.67c
	phenethyl alcohol	10.95	1113	1116	44.92±0.69b	48.58±1.9ab	47.02±4.14ab	48.66±4.69ab	46.06±4.31b	49.55±3.46ab	52.67±0.62a
	cis-3-Nonen-1-ol	12.78	1150	1156	1.92±0.18c	2.33±0.3bc	2.59±0.23ab	2.6±0.23ab	2.83±0.41ab	2.65±0.22ab	3.1±0.45a
	3,6-Nonenylidene-1-ol	12.89	1152	1156	2.33±0.37	2.09±0.18	2.4±0.24	2.33±0.07	2.26±0.3	2.03±0.27	2.46±0.16
	trans-Linalool Oxide (Pyran)	13.87	1172	1173	22.81±0.16b	28.33±2.4a	28.62±3.09a	28.37±4.09a	28.12±2.56a	28.82±2.22a	29.04±1.62a
	L-Menthol	14.01	1175	1175	0.23±0.05a	0.13±0bc	0.16±0.01b	0.15±0.02b	0.11±0.03c	0.12±0c	0.15±0.02bc
	α-Terpineol	14.83	1193	1189	3.46±0.41ab	3.52±0.44ab	3.63±0.4a	3.19±0.28abc	3.45±0.29ab	2.76±0.22c	2.87±0.51bc
	6,6-Dimethyl-dicyclo [3.1.1] hept-2-ene-2-ethanol	14.96	1195	1202	1.8±0.17b	1.96±0.24b	2.04±0.19ab	1.86±0.13b	2.16±0.3ab	1.98±0.06b	2.47±0.47a
	nerolidol	16.32	1220	1228	9.02±1.97b	11.08±0.33a	10.72±0.99ab	10.63±0.81ab	11.21±0.78a	10.73±0.5ab	12.42±1.65a
	Dihydrocarbinol	16.51	1223	1195	0.69±0.03c	1.87±0.55b	1.93±0.26ab	2.01±0.36ab	2.51±0.38a	1.76±0.13b	2.35±0.42ab
	(3Z)-3,7-Dimethyl-3,6-octadien-1-ol	16.60	1225	1240	1.45±0.09c	2±0.26a	2.03±0.28a	1.88±0.2ab	1.99±0.17a	1.52±0.23bc	1.91±0.33ab
	geraniol	18.06	1251	1255	53.92±6.97c	78.55±9.14ab	77.55±1.47b	82.24±3.26ab	83.54±9.64ab	84.47±2.64ab	88.92±2.33a

	8-hydroxylinalool	23.83	1357	1361	0.12±0.02b	0.37±0.09a	0.52±0.17a	0.48±0.07a	0.57±0.13a	0.49±0.06a	0.45±0.15a
	trans-Nerolidol	33.78	1552	1564	5.09±0.42b	6.06±1.26ab	6.29±0.34ab	6.89±0.72a	6.36±0.93ab	5.94±0.51ab	6.52±1.21ab
	cedrenol	35.57	1589	1610	1.43±0.07a	0.48±0.04ef	0.57±0.01de	0.71±0.03c	0.58±0.08d	0.46±0.04f	0.94±0.08b
	ethyl alcohol	36.46	1653	1668	1.31±0.02b	1.46±0.29ab	1.59±0.19a	1.67±0.13a	1.59±0.08a	1.51±0ab	1.71±0.07a
	α-Dehydrol	37.41	1784	1791	2.38±0.17a	1.67±0.05c	1.75±0.13bc	1.92±0.19bc	1.8±0.15bc	1.79±0.15bc	1.95±0.17b
	palm oleinol	41.83	1857	1863	0.31±0.07abc	0.34±0.09ab	0.35±0.05a	0.31±0.01abc	0.24±0.03cd	0.25±0.01bcd	0.17±0.02d
	phytol	44.57	2106	2114	1±0.22abc	1.13±0.2ab	1.18±0.21a	1±0.17abc	0.83±0.12bcd	0.81±0.05cd	0.68±0.16d
Esters	cis-3-Hexenylbutyl ester	14.39	1184	1187	5.2±0.54a	3.71±0.45bc	3.87±0.58b	2.98±0.62cde	3.33±0.16bcd	2.44±0.03e	2.48±0.61de
	Methyl salicylate	14.62	1188	1192	14.05±0.95c	24.07±1.88ab	24.49±1.66a	23.41±0.31ab	23.55±1.74ab	21.91±1.07b	22.32±1.8ab
	Isovaleryl foliate	16.69	1227	1238	7.31±0.34a	5.73±0.85bc	6.17±1.04b	4.71±0.6cd	5.73±0.25bc	4.39±0.2d	4.25±0.58d
	Butyloctanolactone	19.35	1274	1288	0.38±0.05d	0.4±0.04cd	0.52±0.06bcd	0.52±0.17bcd	0.59±0.08abc	0.64±0.12ab	0.78±0.25a
	Theaspirane	20.38	1292	1302	1.06±0.15b	1.27±0.21ab	1.66±0.5ab	1.68±0.4ab	1.79±0.52a	1.42±0.21ab	1.62±0.31ab
	Geranyl formate	20.54	1295	1300	0.37±0.03b	0.58±0.15a	0.59±0.07a	0.64±0.07a	0.64±0.07a	0.6±0.04a	0.71±0.23a
	Methyl geranylate	21.82	1318	1324	5.6±0.3a	4.75±0.4b	4.78±0.16b	4.17±0.27cd	4.49±0.39bc	3.89±0.05d	4.15±0.48cd
	Folyl caproate	24.86	1377	1380	15.7±0.63a	13.77±2.78ab	12.87±1.04bc	11.26±1.83bc	12.91±0.41bc	11.57±0.96bc	11.07±1.37c
	Hexyl caproate	25.17	1382	1384	11.07±0.72a	8.58±1.48b	8.58±0.61b	7.78±0.68bc	8.29±0.55b	8.25±1.42b	6.4±0.23c
	trans-2-Hexenyl hexanoate	25.33	1385	1391	9.92±0.87bc	10.04±0.22bc	11.62±1.34a	10.69±0.69ab	11.48±0.65a	9.81±0.45bc	9.25±0.15c
	coumarin	27.00	1418	1441	1.24±0.17a	1.13±0.19ab	1.21±0.12ab	1.18±0.14ab	1.19±0.19ab	1.14±0.2ab	0.91±0.21b
	Phenethyl butyrate	27.54	1428	1447	2.54±0.26a	1.64±0.18c	2±0.21b	1.69±0.26bc	1.9±0.06bc	1.72±0.04bc	1.75±0.17bc
	Dimethyl phthalate	27.86	1433	1455	10.93±0.31a	9.39±1.4abc	10.14±0.72ab	8.6±0.87bc	9.92±1.19abc	8.91±0.4bc	8.55±0.88c
	Butyldecalactone	30.11	1477	1496	1.54±0.09	1.49±0.23	1.53±0.11	1.53±0.11	1.6±0.13	1.53±0.05	1.66±0.2
	Dihydroactinidiolide	31.67	1507	1532	1.93±0.12a	1.58±0.24b	1.65±0.1b	1.76±0.06ab	1.81±0.13ab	1.65±0.12b	1.92±0.02a

	Benzyl hexanoate	32.94	1534	1547	0.59±0.03c	0.68±0.15bc	0.76±0.1abc	0.81±0.03ab	0.82±0.09ab	0.8±0.03ab	0.91±0.23a
	Folium benzoate	34.13	1559	1570	7.01±0.46a	3.95±0.67b	4.21±0.34b	4.23±0.14b	4.21±0.38b	4.09±0.16b	3.91±0.05b
	Geranyl isovalerate	34.38	1565	1606	1.13±0.07a	0.62±0.03b	0.76±0.03b	0.73±0.08b	0.73±0.13b	0.7±0.08b	0.81±0.21b
	Hexyl benzoate	34.53	1568	1580	5.25±0.46a	1.99±0.09b	2±0.21b	2.06±0.49b	2.1±0.25b	2.05±0.27b	1.94±0.31b
	Caryophyllin	35.47	1587	1581	0.39±0.05b	0.39±0.07b	0.47±0.06ab	0.51±0.05a	0.48±0.05ab	0.46±0.01ab	0.56±0.07a
	Isooctyl salicylate	40.64	1795	1811	1.27±0.33b	1.14±0.3b	1.11±0.09b	2.52±0.09a	1.26±0.23b	1.15±0.23b	1.49±0.04b
	Isopropyl myristate	41.05	1820	1827	1.16±0.35a	0.56±0.03bc	0.31±0.02c	0.65±0.29b	0.56±0.15bc	0.42±0.03bc	0.36±0.12bc
	Methyl palmitate	42.44	1922	1926	1.62±0.09b	1.85±0.19ab	2.03±0.2a	1.87±0.13ab	1.71±0.01b	1.64±0.15b	1.86±0.17ab
Aldehydes	benzaldehyde	6.19	959	962	2.05±0.34b	4.29±0.73a	5.54±0.4a	5.06±0.51a	5±1.13a	4.82±1.39a	4.71±0.82a
	(E,E)-2,4-Heptadienal	7.41	1009	1012	4.06±0.82b	5.44±0.69a	4.93±0.09ab	5.1±0.29a	5.02±0.14a	4.7±0.32ab	5.35±0.86a
	phenylacetaldehyde (PAG)	8.40	1039	1045	15.53±1.11c	32.87±2.04ab	32.97±3.52ab	29±0.32b	31.38±4.54ab	32.5±1.43ab	35.51±0.72a
	E-2-Octenal	8.86	1055	1060	6.53±0.5ab	6.54±0.25ab	6.33±0.34abc	6.05±0.5bc	6.87±0.57a	5.8±0.33c	6.37±0.38abc
	2-2-[2S,5S)-5-Vinyltetrahydro-5-methylfuran-2-yl] propionaldehyde	12.59	1146	1154	1.37±0.23a	0.94±0.12b	1±0.2b	0.88±0.15bc	0.77±0.12bc	0.63±0.06c	0.66±0.13c
	trans-2-Nonenal	13.10	1157	1162	2.2±0.69	2.4±0.53	2.49±0.55	2.42±0.54	2.54±0.42	2.44±0.43	2.4±0.28
	decanal	15.40	1204	1206	2.45±0.39b	2.54±0.18ab	2.65±0.39ab	2.6±0.25ab	2.98±0.24a	2.53±0.24ab	2.81±0.22ab
	β-Cyclocitraldehyde	16.01	1214	1220	14.86±2.02a	10.13±1.2bc	11.69±0.91bc	11.68±0.41bc	12.27±1.7b	9.65±1.17c	11.88±1.33bc
	cis-Citraldehyde	17.07	1233	1240	15.08±1.49a	13.28± 1.56abc	13.38±1.59ab	12.36±0.13bc	13±0.33bc	11.51±0.19bc	11.41±1.09c
	trans-2-Decenal	18.56	1260	1263	2.17±0.06a	1.74±0.14b	2.22±0.13Aa	1.6±0.28b	1.87±0.24ab	1.59±0.07b	2.22±0.46a
	α-Ethylidene-phenylacetaldehyde	18.79	1263	1279	3.43±0.18c	3.34±0.61c	3.75±0.87bc	3.93±0.57bc	4.39±0.92abc	4.81±0.89ab	5.5±0.9a
	citraldehyde	18.84	1264	1276	4.67±0.7c	7.57±0.07b	8±0.43ab	7.68±0.62b	8.28±0.63ab	7.58±0.29b	8.79±0.46a

	2-Undecenal	24.01	1360	1367	3.19±0.7	3.02±0.29	3.43±0.55	2.94±0.21	3.25±0.21	2.95±0.07	3.75±0.82
	coccaldehyde (CH20)3	29.77	1471	1786	0.76±0.03bc	0.61±0.09c	0.79±0.13bc	0.8±0.13bc	0.89±0.12b	0.89±0.03b	1.23±0.23a
Ketones	ketamine	24.57	1371	1386	0.54±0.17b	1.09±0.13ab	1.33±0.2a	1.54±0.37a	1.59±0.56a	1.63±0.24a	1.51±0.54a
	α-Violanone	26.66	1411	1426	1.46±0.07b	1.35±0.29b	1.52±0.21ab	1.54±0.14ab	1.66±0.15ab	1.48±0.06b	1.91±0.47a
	Geranylacetone	28.01	1437	1453	2.44±0.34a	1.85±0.21bc	2.05±0.26bc	1.92±0.05bc	2.15±0.11ab	1.83±0.07bc	1.78±0.11c
	β-Violanone	29.48	1465	1486	9.34±0.14b	8.96±1.26b	9.96±0.96b	10.42±0.65ab	10.67±0.85ab	10.24±0.4ab	12.06±2.26a
	4-[2,2,6-Trimethyl-7-oxabicyclo [4.1.0]hept-1-yl]-3-buten-2-one	29.64	1468	1473	3.28±0.18a	2.14±0.26c	2.35±0.36c	2.28±0.09c	2.45±0.31bc	2.14±0.16c	2.85±0.4ab
Hydrocarbons	limonene	7.97	1026	1030	30.64±1.76	31.63±0.98	32.12±1.7	32.78±0.77	34.91±0.42	31.85±5.77	31.47±3.9
	naphthalene	14.20	1180	1182	6.79±0.19a	3.14±0.27e	3.55±0.34de	3.81±0.43d	6.08±0.53b	4.09±0.11d	5.09±0.43c
	dodecane	15.14	1199	1200	1.09±0.08ab	1.03±0.12ab	1.11±0.12ab	0.88±0.11b	1.07±0.17ab	1.29±0.22a	1.11±0.17ab
	(E)-4-Decen-6-yne	19.53	1277	1244	0.71±0.06c	1.04±0.18b	1.07±0.08b	1.17±0.08ab	1.17±0.25ab	1.05±0.16b	1.45±0.24a
	1-Methylnaphthalene	20.10	1286	1307	4.51±0.34a	2.72±0.1c	3.34±0.51b	3.38±0.34b	4.57±0.39a	3.21±0.22bc	3.68±0.08b
	tridecane	20.76	1299	1300	1.77±0.08	1.74±0.31	1.86±0.32	1.87±0.27	1.89±0.27	2±0.35	2.09±0.18
	Picrasidine	22.97	1341	1389	1.57±0.14a	0.98±0.09b	1.14±0.09b	1.08±0.22b	1.03±0.08b	0.99±0.15b	1.08±0.14b
	n-tetradecane	25.90	1396	1400	1.77±0.3a	1.06±0.11c	1.21±0.11bc	1.2±0.1bc	1.61±0.09ab	1.31±0.02bc	1.85±0.59a
	Δ - juniperene	31.52	1504	1524	3.17±0.14a	2.33±0.4b	2.38±0.14b	2.32±0.17b	2.22±0.15b	2.05±0.08b	2.25±0.21b
	β - curcumene	32.18	1518	1514	1.24±0.05	1.23±0.27	1.3±0.18	1.33±0.14	1.28±0.16	1.23±0.13	1.27±0.09
Carboxylic acids	trans-3-Hexenoic acid	7.23	1002	1021	4.18±0.04d	8.57±0.23a	8.33±0.98a	6.19±0.37bc	7.47±0.97ab	6.07±0.57c	6.86±1.2bc
	heptanoic acid	9.63	1075	1078	1.36±0.26	1.65±0.05	1.68±0.14	1.46±0.27	1.51±0.24	1.62±0.56	1.66±0.21
	(2E)-3,7-Dimethyl-2,6-octadienoic acid	23.61	1353	1344	7.65±1.01b	16.08±3.72a	16.33±2.36a	15.98±2.34a	15.97±3.84a	15.55±3.43a	15.93±3.14a

	(Z)-7-Decen-5-oic acid	29.94	1473	1518	1.16±0.1c	1.3±0.29abc	1.68±0.38a	1.65±0.3ab	1.48±0.18abc	1.27±0.09bc	1.41±0.05abc
Phenols	eugenol	23.22	1345	1357	1.92±0.07	1.85±0.25	2.15±0.11	2.13±0.17	1.92±0.23	2.32±0.55	2.13±0.54
	2,6-Di-tert-butyl-4-methylphenol	30.82	1490	1513	0.48±0.04a	0.24±0.04b	0.28±0.05b	0.32±0.07b	0.32±0.08b	0.32±0.04b	0.32±0.09b
	2,4-Di-tert-butylphenol	31.10	1495	1519	4.91±0.06a	3.02±0.11b	3.28±0.33b	3.34±0.72b	3.43±0.68b	3.47±0.63b	4.79±0.75a

Note: Different letters indicate significant differences （$p<0.05$）. R. T. – Retention time; RI- Retention Index

Table S5. Relative content of main non-volatile components during different rolling times of black tea (mg/g).

Compound name	Theoretical m/z	MS/MS fragments	Relative content（mg/g）						
			RT0	RT20	RT40	RT60	RT80	RT100	RT120
Theobromine	181.0720	163,138,122,110,83,69,67,56	0.52±0.027ab	0.55±0.027a	0.54±0.008ab	0.51±0.017b	0.55±0.02a	0.55±0.033a	0.56±0.035a
Theophylline	181.0720	124,96,69	0.99±0.076	1.02±0.014	1±0.046	0.97±0.032	1±0.013	0.99±0.079	0.94±0.077
Caffeine	195.0877	138,123,110,83,69,56	30.43±0.545c	34.71±0.645b	36.17±0.278a	35.88±0.171a	35.81±0.489a	36.03±0.769a	36.09±0.854a
Theaflavin	565.1341	427277139.0000	0.08±0.001f	0.14±0.004a	0.13±0.005b	0.12±0.003c	0.11±0.001cd	0.11±0.004d	0.1±0.002e
Theaflavin-3-gallate	717.1450	579379277139.0000	0.07±0.002d	0.13±0.001ab	0.13±0.0008a	0.12±0.002ab	0.12±0.001ab	0.12±0.003b	0.12±0.003c
Theaflavin-3'-gallate	717.1450	591579333277139.0000	0.09±0.002c	0.14±0.005a	0.13±0.001b	0.13±0.006b	0.13±0.007ab	0.12±0.007b	0.12±0.007b
Theaflavins-3,3'-digallate	869.1560	743699529333277000.0000	0.2±0.008b	0.24±0.011a	0.24±0.006a	0.25±0.009a	0.24±0.005a	0.24±0.005a	0.24±0.007a
Shikimic acid	175.0601	158,84,56	10.9±0.638a	9.23±0.3b	9.28±0.49Bb	9.25±0.168Bb	8.9±0.179b	8.94±0.126b	8.92±0.094b
Tartaric acid	151.0147	119, 90, 75	0.33±0.008a	0.25±0.01b	0.21±0.001Cc	0.2±0.005Cd	0.18±0.006e	0.16±0.002f	0.16±0.004f
α-Ketoglutaric acid	145.0426	129, 101, 87	0.23±0.014b	0.25±0.011a	0.23±0.01b	0.24±0.008ab	0.23±0.002b	0.24±0.005b	0.24±0.005ab
Succinic acid	101.0180	85, 57	0.04±0.002	0.04±0.001	0.04±0	0.04±0	0.04±0	0.04±0	0.04±0
Gallic acid	171.0288	153,135,127,125,109,107,81,53	0.13±0.009e	0.17±0.006a	0.16±0.009ab	0.15±0.004bc	0.15±0.003c	0.14±0.005d	0.14±0.002d
Chlorogenic acid	355.1042	163,145,135,117,89	0.02±0a	0.01±0b	0.01±0c	0±0d	0.01±0c	0±0e	0±0f

Salicylic acid	139.0390	121,93,65	0.46±0.008a	0.1±0.007b	0.08±0.005c	0.06±0.003d	0.05±0.001e	0.04±0.002f	0.04±0.002f
Pyruvic acid			0.02±0.001ab	0.02±0.001a	0.02±0.001a	0.02±0ab	0.02±0ab	0.02±0.001b	0.02±0ab
Vitexin	433.1129	415,397,367,313,283	0.04±0.002a	0.03±0.001b	0.03±0.001b	0.03±0.001b	0.03±0b	0.03±0b	0.03±0b
Myricetin	319.0448	273,245,217,165,153,137,111,69	0.17±0.009a	0.07±0.003b	0.06±0.003c	0.05±0.001d	0.04±0.002d	0.03±0.001e	0.03±0.001e
Quercetin	449.1078	303,129,85,71,57	0.16±0.007b	0.18±0.007a	0.17±0.007a	0.17±0.003a	0.16±0.006b	0.15±0b	0.15±0.004b
Kaempferol	287.0550	153,69	0.29±0.023c	0.33±0.021ab	0.34±0.019a	0.3±0.013bc	0.34±0.018a	0.32±0.005abc	0.32±0.013abc
Myricetin 3-O-galactoside	465.1028	303	0.36±0.021a	0.14±0.009b	0.13±0.007c	0.09±0.005d	0.08±0.005d	0.07±0.003e	0.07±0.003e
Glucosyl-vitexin	611.1607	449,329,287,85	0.03±0.002bc	0.04±0.001a	0.03±0e	0.04±0.001a	0.03±0.001b	0.03±0cd	0.03±0.001de
Quercetin-7-O-β-D-glucopyranoside	465.1028	303	0.08±0.007	0.08±0.005	0.08±0.001	0.08±0.001	0.08±0.003	0.08±0.001	0.08±0.001
Quercetin-3-o-rutinose	611.1607	449,329,287,85	0.29±0.017a	0.22±0.008b	0.23±0.013b	0.2±0.006c	0.2±0.006c	0.18±0.01c	0.19±0.006c
vitexin-2″-o-rhamnoside	579.1708	415,397,367,313,283,85	0.05±0.002c	0.06±0.002b	0.04±0.002c	0.06±0.001b	0.06±0.001b	0.06±0.002a	0.07±0.002a
Quercitrin	449.1078	303,129,85,71,57	0.22±0.006d	0.23±0.008d	0.25±0.007bc	0.26±0.009bc	0.28±0.004a	0.26±0.012ab	0.25±0.015c
Kaempferitrin	579.1708	433,287,85	0.06±0.001a	0.05±0.001c	0.04±0.001d	0.05±0c	0.05±0.001c	0.05±0.002b	0.05±0.002b
Quercetin-7- O-α-L-rhamnoside	449.1078	303,229,85	0.08±0.007	0.08±0.005	0.08±0.001	0.08±0.001	0.08±0.003	0.08±0.001	0.08±0.001
Dihydrokaempferol 7-O-rhamnoside	435.1286	400,332,290,272,244,195,129,107,85,57	0.03±0.001a	0.03±0.001b	0.03±0.001b	0.02±0.001b	0.03±0.001b	0.03±0.001b	0.03±0.001b
Quercetin-3-galactoside	463.0866	319, 317, 301	0.05±0.002c	0.06±0.002ab	0.06±0.002b	0.06±0.001a	0.05±0.001c	0.05±0c	0.05±0.002c

Isovitexin 2''-O-arabinoside	565.1552	435,369,314,115,73	0.25±0.017d	0.24±0.009d	0.26±0.008cd	0.26±0.013Bcd	0.28±0.007ab	0.29±0.012a	0.27±0.01bc
Benzyl β-glucosidea	293.0996	265,234,205,184,169,143,129,117	0.1±0.002a	0.1±0.007a	0.1±0.001a	0.09±0.005b	0.09±0.004b	0.08±0.002b	0.08±0.003b
2-Phenylethyl glucosidea	307.1152	292,266,232,215,201,185,165,144,126,102,85	1.63±0.048a	0.14±0.012b	0.1±0.006c	0.1±0.005c	0.07±0.007d	0.05±0.005d	0.05±0.004d
(Z)-3-hexenyl glucosidea	285.1309	268,235,211,179,153,129,105,92	0.02±0a	0.02±0b	0.01±0c	0.01±0d	0.01±0e	0.01±0e	0.01±0e
Geranyl glucosidea	339.1778	301,262,236,203,179,164,117	0.04±0a	0.03±0b	0.02±0c	0.02±0c	0.02±0c	0.02±0d	0.02±0cd
2-Phenylethyl primeveroside	439.1575	401,307,275,218,170,133	0.09±0.009a	0.03±0.001c	0.03±0.001bc	0.03±0.001bc	0.03±0bc	0.03±0.001b	0.03±0.001bc
(Z)-3-hexenyl primeveroside	417.1731	417,375,336,317,301,285,242,217,195	0.04±0.002	nd	nd	nd	nd	nd	nd
Geranyl primeverosidea	471.2201	335,275,245,203,169,123	0.19±0.012a	0.01±0b	0.003±0b	nd	nd	nd	nd
L-Aspartic acid	134.0448	88,74,61	0.09±0.007b	0.09±0.003ab	0.09±0.004ab	0.1±0.004a	0.09±0.004ab	0.09±0.004ab	0.09±0.004ab
L-Asparagine	133.0608	87,74,70,60	0.07±0.004a	0.06±0.001b	0.06±0.001b	0.06±0.001b	0.06±0b	0.06±0.001b	0.06±0b
L-Glutamic acid	148.0604	130,102,84,56	0.11±0.009	0.11±0.002	0.11±0.006	0.11±0.005	0.11±0.004	0.11±0.005	0.11±0.005
L-Glutamine	147.0764	130,84,56	0.1±0.006a	0.08±0.005c	0.09±0.003bc	0.09±0.003bc	0.09±0.005b	0.09±0.002b	0.09±0.004b

L-Lysine	147.1128	102,84,56	0.05±0a	0.04±0.002ab	0.04±0.002b	0.04±0.002bc	0.04±0.002c	0.04±0.002c	0.04±0.002c
L-Threonine	120.0655	94,74,56	1.34±0.085a	1.07±0.064b	1.01±0.053bc	1.02±0.028bc	0.96±0.016c	0.98±0.027c	0.96±0.038c
L-Hisidine	156.0768	110,93,83,56	0.04±0a	0.03±0.001b	0.03±0c	0.02±0.001d	0.02±0e	0.02±0f	0.02±0f
L-Proline	116.0706	70	0.46±0.029a	0.42±0.014b	0.42±0.012b	0.43±0.015b	0.42±0.011b	0.43±0.014b	0.43±0.01b
L-Valine	118.0863	72,55	0.68±0.046	0.67±0.018	0.67±0.024	0.67±0.011	0.66±0.011	0.66±0.005	0.65±0.006
L-Theanine	175.1077	158,129,84,56	10.47±1.068	10.05±1.681	10.06±1.551	10.06±1.634	9.84±1.841	9.82±1.784	9.66±1.493
L-Tyrosine	182.0812	136,123,119,95,91,27	0.59±0.037a	0.54±0.008b	0.54±0.015bc	0.53±0.014bcd	0.52±0.01bcd	0.51±0.016cd	0.5±0.012d
L-Leucine	132.1019	86,69,55	5.78±0.108a	5.39±0.198b	5.18±0.145cd	5.24±0.076bc	5.1±0.04cd	5.09±0.117cd	5±0.116d
L-Phenylalanine	166.0863	120,103,77,51	0.17±0.027e	0.36±0.027d	0.43±0.046c	0.48±0.038b	0.5±0.026ab	0.53±0.046ab	0.54±0.032a
L-Tryptophan	205.0972	188,170,146,118	1.33±0.022a	1.17±0.038b	1.14±0.041bc	1.13±0.066bc	1.07±0.06cd	1.07±0.053cd	1.03±0.044d
L-Methionine	150.0583	56, 61, 74, 87, 104, 115, 133	0.02±0.001cd	0.02±0a	0.02±0b	0.02±0c	0.02±0d	0.01±0e	0.01±0f
γ-aminobutyric acid	104.0707	83, 75, 71	0.03±0.001	0.03±0.001	0.03±0	0.03±0	0.03±0	0.03±0.001	0.03±0
Epigallocatechin gallate/EGCG	459.0922	289,205,181,153,139,123,65	2.17±0.071a	0.45±0.019b	0.39±0.029c	0.3±0.013d	0.24±0.023e	0.18±0.013f	0.18±0.014f
Gallocatechin gallate/GCG	459.0922	289,205,181,153,139,123,65	3.2±0.056a	0.73±0.04b	0.61±0.029c	0.48±0.018d	0.38±0.031e	0.29±0.024f	0.29±0.024f
Epicatechin gallate/ECG	443.0973	291,273,207,153,139,77	2.43±0.091a	1.24±0.036b	1.06±0.063c	0.8±0.032d	0.67±0.053e	0.49±0.028f	0.49±0.028f
Epicatechin /EC	291.0863	207,139,123,95,55	0.93±0.034a	0.25±0.003b	0.18±0.001c	0.12±0.005d	0.1±0.01d	0.07±0.001e	0.07±0.001e
Epigallocatechin/EGC	307.0812	223,195,163,139,95	4.22±0.086a	0.36±0.017b	0.28±0.012c	0.21±0.009d	0.16±0.008de	0.12±0.008e	0.12±0.006e

gallocatechin/GC	307.0812	223,195,163,139,95	0.17±0.011a	0.12±0.006c	0.13±0.004b	0.14±0.009b	0.13±0.007b	0.13±0.005bc	0.14±0.007b
(+)-Catechin/C	291.0863	207,139,123,95,55	1.43±0.089a	0.36±0.016b	0.26±0.009c	0.17±0.003d	0.15±0.006de	0.1±0.006e	0.1±0.006e

Note: Different letters indicate significant differences (p<0.05). nd= not detected
